# Zastosowanie Wemurafenibu w Opornej Na Leczenie Histiocytozie z Komórek Langerhansa

**DOI:** 10.34763/devperiodmed.20182204.376378

**Published:** 2019-01-14

**Authors:** Anna Raciborska, Zofia Małas, Andrzej Tysarowski

**Affiliations:** 1 Klinika Onkologii i Chirurgii Onkologicznej Dzieci i Młodzieży, Instytut Matki i Dziecka, Warszawa, Polska; 2 Zakład Patologii i Diagnostyki Laboratoryjnej oraz Zakład Onkologii Molekularnej i Translacyjnej, Centrum Onkologii – Instytut im. Marii Skłodowskiej-Curie, Warszawa, Polska

**Keywords:** histiocytoza z komórek Langerhansa, inhibitory BRAF, wemurafenib, Langerhans histiocytosis, BRAF inhibitors, vemurafenib

## Abstract

Histiocytoza z komórek Langerhansa (LCH) jest rzadką chorobą, u podłoża której może leżeć występowanie zaburzeń w szlaku kinaz aktywowanych mitogenami (MAPK). Obecnie wiadomo, że występowanie powyższych nieprawidłowości łączy się zazwyczaj z bardziej agresywną formą choroby, częstszą opornością na konwencjonalną chemioterapię, a także z większym prawdopodobieństwem wznowy oraz progresji. Od niedawna istnieje możliwość zastosowania w leczeniu pacjentów z LCH inhibitorów BRAF, jednak nie ma obecnie jasnych wytycznych co do kryteriów włączenia powyższej terapii. Niewiele jest też danych literaturowych dotyczących bezpieczeństwa stosowania tego typu preparatów u małoletnich pacjentów. W pracy prezentujemy dziewczynkę z ciężką postacią histiocytozy z komórek Langerhansa niereagującą na leczenie konwencjonalne, u której ustąpienie objawów choroby uzyskano dopiero po włączeniu wemurafenibu. Przypadek ten pokazuje konieczność poszerzania diagnostyki również o badania molekularne oraz możliwość zastosowania leczenia celowanego w tej grupie pacjentów.

## Abstract (en)

Langerhans Cell Histiocytosis (LCH) is a rare disease involving the occurrence of disturbances in the mitogene-activated kinases pathway (MAPK). At present, it is known that the appearance of such disorders is usually connected with a more aggressive form of the disease, more frequently resistant to conventional chemotherapy, as well as characterised by a higher probability of relapse and progression. Since recently, it has been possible to apply BRAF inhibitors in the treatment of LCH patients, however, there are no clear guidelines regarding the criteria for implementing this type of therapy. There are very little literature data as to the safety of using such formulations among juvenile patients. In this paper we present a girl with severe Langerhans Cell Histiocytosis, not responding to conventional treatment, who only after adding vemurafenib achieved a regression of symptoms. This case shows the necessity to expand the diagnostics with molecular tests and the possibility of applying targeted treatment for patients of this kind.

## Wstęp

Histiocytoza z komórek Langerhansa (LCH) jest rzadką chorobą, która może występować w postaci jednej zmiany lub obejmować swoim zasięgiem jednocześnie wiele regionów ciała. Stwierdza się ją z częstością od 0,1 do 1 nowych zachorowań na 100 000 dzieci na rok. Najczęściej rozpoznawana jest u pacjentów do 6 r.ż. Przyczyna choroby nie jest do końca poznana. U jej podłoża leży klonalna proliferacja histiocytów (tzw. komórek Langerhansa; LC) immunohistochemicznie oraz morfologicznie przypominających komórki dendrytyczne. Spektrum objawów klinicznych jest szerokie, od stwierdzanych pojedynczych ognisk do objawów z wielu narządów czy układów mogących zagrażać życiu. Obserwowane symptomy choroby są wynikiem zarówno akumulacji nieprawidłowych komórek w tkankach i narządach, jak i toczącego się procesu zapalnego [1, 2, 3, 4, 5]. W 2010 r. Badalian-Very opublikował pracę, w której opisał obecność mutacji w obrębie genu BRAF ^V600E^ w histiocytach u pacjentów z objawami LCH [6]. Obecnie wiadomo, że u wielu chorych można wykryć mutacje w szlaku kinaz MAP w komórkach dendrytycznych. Szczególnie często, bo ok. 60 % dotyczy to mutacji w obrębie genu BRAF [7, 8, 9]. Rokowanie u pacjentów, u których wykrywa się powyższe mutacje jest gorsze, nierzadko stwierdza się u nich zajęcie tzw. narządów ryzyka (układ krwiotwórczy, wątroba, śledziona). Również nawroty i reaktywacje choroby są zdecydowanie częstsze w tej grupie chorych [2, 3, 4]. Chociaż terapia konwencjonalna jest podstawą leczenia LCH, to obecnie coraz częściej wykorzystuje się w nim również inhibitory BRAF m.in wemurafenib – drobnocząsteczkowy, celowany lek przeciwnowotworowy, którego mechanizm działania polega na hamowaniu kinazy seroninowo-treoninowej BRAF [10, 11, 12]. Jednak u dzieci z LCH nie jest określony ani czas trwania, ani intensywność leczenia. Niesprecyzowane są również kryteria wymagane do włączenia takiej terapii.

## Cel pracy

Celem niniejszego opracowania jest pokazanie możliwości zastosowania wemurafenibu w opornej na leczenie histiocytozie z komórek Langerhansa w oparciu o badanie molekularne. Wg naszej wiedzy jest to pierwszy przypadek wykorzystania tego typu terapii u małoletniego pacjenta z LCH w Polsce i jeden z nielicznych na świecie.

## Opis przypadku

8-miesięczną dziewczynkę przyjęto do szpitala z powodu swędzących, uogólnionych zmian skórnych, o niejasnej etiologii, niepoddających się leczeniu objawowemu (leki przeciwalergiczne, maści sterydowe/cholesterolowe, dieta eliminacyjna) stwierdzanych od 3 miesiąca życia dziecka. Wywiad rodzinny w kierunku chorób nowotworowych był nieistotny. Na podstawie przeprowadzonych badań (analiza krwi, biopsja skóry oraz badania obrazowe) rozpoznano wieloukładową postać LCH bez zajęcia organów ryzyka (zajęcie skóry, pojedyncze ognisko w kości piszczelowej). Włączono leczenie zgodnie z protokołem LCH III (prednizon oraz winblastyna). Mimo stosowanego leczenia stan dziecka się nie poprawiał. Kolejne badania wykazały narastanie zmian w układzie kostnym. Utrzymywały się uogólnione zmiany skórne. W ramach poszerzania diagnostyki wykonano badanie PET-CT oraz MR całego ciała, które wykazały liczne zmiany w układzie kostnym. Dodatkowo przeprowadzono badanie molekularne. Stwierdzono obecność mutacji w kodonie 600 genu BRAF zarówno w zmienionej chorobowo tkance (bloczek parafinowy), jak i we krwi obwodowej, w której oceniano status genu BRAF na podstawie analizy molekularnej wolnokrążącego DNA uwalnianego z komórek zmienionych chorobowo (ctDNA/cfDNA – ang. circulating tumor DNA / cell free DNA). Zmodyfikowano terapię, włączono do leczenia arabinozyd cytozyny oraz winkrystynę, ale nie uzyskano poprawy. Kolejnym zastosowanym lekiem była kladrybina. W badaniu PET-CT po 2. cyklu leczenia stwierdzono progresję metaboliczną zmian w kościach. Ponadto, w badaniu klinicznym oraz badaniach laboratoryjnych, pojawiły się objawy zajęcia tzw. narządów ryzyka (wątroba, śledziona, układ krwiotwórczy). Wobec braku reakcji na dotychczasowe leczenie, po uzyskaniu zgody Komisji Bioetycznej, dziewczynce włączono wemurafenib w dawce 10 mg/kg 2 razy na dobę uzyskując w szybkim czasie ustąpienie objawów choroby. Leczenie prowadzono monitorując status genu BRAF na podstawie molekularnej analizy cfDNA. W ciągu całego okresu nie obserwowano toksyczności zastosowanego leczenia. Dziewczynka pozostaje w całkowitej remisji choroby zasadniczej.

## Dyskusja

Choroby z kręgu histiocytoz to rzadkie schorzenia o różnorodnym obrazie i przebiegu klinicznym, których patogeneza związana jest z zaburzeniem proliferacji i różnicowania komórek układu fagocytarnego. Ze względu na klonalny charakter rozrostów zaliczane są do chorób nowotworowych, mimo że czasami przebieg kliniczny sugeruje inny mechanizm patogenetyczny. Obecna klasyfikacja dzieli zaburzenia z kręgu histiocytoz na 5 grup. Histiocytoza z komórek Langerhansa należy do grupy L razem z chorobą Erdheima-Chestera (ECD), indeterminate cell histiocytosis (ICH), pozaskórną młodzieńczą postacią ksantogranuloma (extra-cutaneus JXG), jak i mieszanymi postaciami LCH i ECD [1]. U dzieci i młodzieży najczęściej występuje histiocytoza z komórek Langerhansa. Patogeneza LCH nie jest do końca poznana. Możliwość wystąpienia samoistnych remisji oraz łagodny niekiedy przebieg kliniczny może sugerować klonalny rozrost reaktywny, a nie nowotworowy. Istnieją teorie, według których zasadniczą rolę w powstaniu choroby odgrywa wcześniejsza dysregulacja układu immunologicznego i reakcja zapalna, a także proliferacja wtórnych komórek LC po ekspozycji na nieokreślony do końca bodziec. W ostatnich latach odkryto, że u wielu pacjentów nadmierna „reakcja zapalna” wynika z występowania mutacji w szlaku kinaz MAP w komórkach dendrytycznych [6, 7, 8, 9].

Przebieg LCH może być różnorodny, czasami bywa nieprzewidywalny. Waha się od spontanicznej regresji, poprzez wieloletni okres zaostrzeń choroby, do agresywnej postaci w szybkim tempie prowadzącej do śmierci. Obecnie wiadomo, że występowanie mutacji w genie BRAF łączy się zazwyczaj z bardziej agresywną formą choroby, częstszą opornością na konwencjonalną chemioterapię, a także z większym prawdopodobieństwem wznowy oraz progresji [2, 3, 4].

Określenie konkretnych zmian w materiale genetycznym będących przyczyną powstających zaburzeń stało się podstawą do poszukiwania możliwości leczenia celowanego. Wykorzystując wyniki leczenia innego nowotworu, gdzie stwierdzono podobne zaburzenia (czerniak), podjęto próbę zastosowania inhibitorów BRAF również u pacjentów z LCH uzyskując ustąpienie oznak choroby w przeciągu kilku tygodni [5, 10, 11, 12]. Pierwszym zastosowanym preparatem był wemurafenib, selektywny inhibitor kinazy seroninowo-treoninowej BRAF (blokuje on kinazę BRAF^V600E^, co w rezultacie prowadzi do zahamowania cyklu komórkowego). U naszej pacjentki wykryto charakterystyczną mutację i na tej podstawie, w oparciu o niekorzystny przebieg kliniczny oraz nieliczne dane literaturowe, podjęto decyzję o wdrożeniu leczenia wemurafenibem. Po kilku tygodniach uzyskano ustąpienie objawów choroby bez ujemnych wczesnych skutków zastosowanego leczenia.

Obecnie nie jest dokładnie określony ani okres trwania leczenia, ani odległe skutki terapii. Podejmowane są próby zarówno zwiększenia czasu leczenia preparatem, jak i ponownego włączania konwencjonalnej chemioterapii, jako leczenia podtrzymującego remisję. Niestety u części pacjentów nie stwierdza się reakcji na zastosowane leczenie lub obserwuje się nawrót choroby. Jedną z przyczyn może być rozwijanie się oporności na lek lub pierwotna oporność, która występuje u niewielkiego odsetka chorych.

Otwarte pozostaje również pytanie czy pacjentów, u których nie stwierdza się klinicznych oznak choroby, można uznać za wyleczonych i czy przypadkiem leczenie nie powinno być stosowane do czasu uzyskania ujemnych wyników stwierdzanych mutacji w szlaku kinaz MAP we krwi chorego, na podstawie monitorowania molekularnego statusu mutacji genu BRAF w wolnokrążącym DNA.

Oczywiście powyższe opracowanie nie wyczerpuje podjętego tematu, mamy jednak nadzieję, iż praca uzasadnia konieczność analizy molekularnej, u każdego pacjenta z LCH, a także pomoże podjąć decyzję dotyczącą zastosowania wemurafenibu u dzieci z oporną na leczenie LCH, u których wykryto mutację w genie BRAF. W chwili obecnej, w Klinice Onkologii i Chirurgii Onkologicznej Dzieci i Młodzieży IMID u wszystkich pacjentów z potwierdzonym rozpoznaniem LCH, wykonywane jest badanie molekularne z oznaczeniem mutacji w genie BRAF, aby w razie zaistnienia takiej konieczności, zapewnić możliwość leczenia celowanego.
